# Theoretical conditions for the coexistence of viral strains with differences in phenotypic traits: a bifurcation analysis

**DOI:** 10.1098/rsos.181179

**Published:** 2019-01-09

**Authors:** Anel Nurtay, Matthew G. Hennessy, Josep Sardanyés, Lluís Alsedà, Santiago F. Elena

**Affiliations:** 1 Centre de Recerca Matemàtica, Universitat Autònoma de Barcelona, Campus de Bellaterra, Edifici C, 08193 Bellaterra, Spain; 2 Barcelona Graduate School of Mathematics (BGSMath), Universitat Autònoma de Barcelona, Campus de Bellaterra, Edifici C, 08193 Bellaterra, Spain; 3 Departament de Matemàtiques, Universitat Autònoma de Barcelona, Campus de Bellaterra, Edifici C, 08193 Bellaterra, Spain; 4 Instituto de Biología Integrativa de Sistemas, CSIC-Universitat de València, Parc Científic UV, Paterna, València 46980, Spain; 5 Santa Fe Institute, 1399 Hyde Park Road, Santa Fe, NM 87501, USA

**Keywords:** bifurcations, epidemiology, infection dynamics, mathematical biology, virus evolution

## Abstract

We investigate the dynamics of a wild-type viral strain which generates mutant strains differing in phenotypic properties for infectivity, virulence and mutation rates. We study, by means of a mathematical model and bifurcation analysis, conditions under which the wild-type and mutant viruses, which compete for the same host cells, can coexist. The coexistence conditions are formulated in terms of the basic reproductive numbers of the strains, a maximum value of the mutation rate and the virulence of the pathogens. The analysis reveals that parameter space can be divided into five regions, each with distinct dynamics, that are organized around degenerate Bogdanov–Takens and zero-Hopf bifurcations, the latter of which gives rise to a curve of transcritical bifurcations of periodic orbits. These results provide new insights into the conditions by which viral populations may contain multiple coexisting strains in a stable manner.

## 1. Introduction

The combination of very large population sizes, very short generation times, and lack of proof-reading mechanisms during genome replication confer viral populations with an extremely high evolutionary plasticity that allow them to quickly adapt to environmental changes such as new host species, the presence of antiviral drugs, new transmission routes or to new vectors [1]. This tremendous evolvability, coupled with densely populated animal and plant susceptible hosts (in many cases lacking genetic variability for resistance to infection), are the reasons for the persistence and emergence of new viral diseases or the re-emergence of new strains with novel properties of already known diseases. The continuous emergence of new mutants leads to an overlap in existence of wild-type (hereafter referred to as wt) and mutant genotypes of the same virus within individual infected hosts [2,3]. This cloud of mutants is usually known as a viral quasi-species [1] and it constitutes the target of selection, instead of the individual viral genomes. This reservoir of coexisting genetic variants may lead to the emergence of new genotypes with different host ranges, pathologies and epidemiological properties that may result in outbreaks [4,5].

With the development of high-coverage, ultra-deep sequencing techniques, it is now possible to characterize in great detail virus genetic diversity along the course of infection of individual hosts, demonstrating the coexistence of multiple mutant genotypes within individual hosts, some even during long periods of time [6–8]. Furthermore, some of these studies have also shown that dynamics are highly complex and do not only depend on the differences in replicative fitness among individual genotypes, but on other parameters such as the size and frequency of within-host bottlenecks, complementation of strains, fixation of additional mutations on the same genotype, epistasis, the availability of beneficial mutations (which indeed depends on the degree of adaptation to the host), the load of deleterious mutations or clonal interference among coexisting beneficial mutations [9–12].

What evolutionary mechanisms determine the long-term coexistence of different genetic variants and strains that, in principle, shall be competing for the same resources (e.g. target cells)? Understanding the evolutionary forces of such intraspecific competition or strain coexistence are essential for understanding the long-term fate and composition of viral populations and for a thoughtful design of more robust control strategies for known and future outbreaks [13]. Consequently, the coexistence of evolving pathogens has been the target of extensive research [14–17].

In the mathematical theory of population genetics, mutation, which is understood as any change in the genome of an organism, is often modelled as a ‘flow’ between populations of initial wt individuals and emerging mutant individuals [18–21]. In the present article, we introduce mutation into a mathematical model in a similar way although avoiding forceful restrictions put upon mutant strains and allowing the mutant virus to have similar characteristics to the wt virus. The difference between mutant and wt strains occurs when focusing the study on specific phenotypic characteristics. A number of studies employ fitness to investigate the survival of a population in dynamical systems that present competition or coexistence [22–24]. As an accumulative property resulting from various phenotypic traits, fitness is a convenient measure of the overall success of the population. In the case of virus evolution, fitness can be considered proportional to the infection rate of the virus [25–27], and measured by the infection rate when other parameters are fixed. It is known that infection rates differ between strains, a fact which has significant implications for the evolution of virulence and strain coexistence in nature [28]. Even the balance between genetic diversity and competition is believed to be achieved due to the possibility of coexistence among strains with differences in infection rates [29]. In other words, a direct competition for infecting available cells mediates the stable coexistence only when competitive abilities in viral clones satisfy certain pairwise asymmetries [30].

A secondary phenotypic characteristic that can differ between viral strains is their strategy for exploiting the host cell, i.e. their virulence [31]. The evolution of virulence has received great attention from theoreticians, particularly on the coevolution between resistance and virulence traits and their combined effect on host and virus dynamics [32,33]. However, understanding the evolution of virulence for coexisting viral strains still requires attention due to the complexity of the underlying evolutionary and dynamical processes, being inherently nonlinear. Many of the models brought forward to explain the evolution of virulence take into consideration the processes of coinfection and superinfection [34–37], where the host or the host cell is infected simultaneously by more than one pathogen particle (coinfection) or sequentially by different pathogens (superinfection). Here, infection of a cell will be modelled with virions of a single strain and superinfection will be neglected. The benefit of this approach is that analytical insights into coexistence of viral strains can be obtained.

The purpose of this paper is to illustrate, by means of a dynamical mathematical model, the conditions for coexistence of viral strains that considers both a wt viral strain and its mutants. We present analytical and numerical results focusing on the parameters related to the differential phenotypic traits of the wt and mutant strains. Conditions for coexistence and invasion have previously been studied using a mathematical model of one host shared between two competing parasites [38]. However, this model did not incorporate the mutation of parasites as a factor, and thus neglected the input of new strains into the system. Nearly all mathematical models in epidemiology detecting various dynamical behaviours with multi-strain infections illustrate the necessity of numerical approaches and the dependency of such models on a large number of parameters [39–43]. A classical approach used in epidemiology [44] to circumvent this difficulty is to introduce dimensionless parameter groups, such as the basic reproduction number *R*_0_, as in [45]. However, the number of dimensionless groups can still become large as additional complexity is introduced into the model, as is the case here. Thus, we perform a bifurcation analysis to systematically track how the dynamics of the system change as multiple parameters are varied. In general, the study of parameters in terms of their effect on the stability of certain states of a model is a highly effective way to gain important insights into the investigated system [46–48].

The paper is organized as follows. We first introduce a detailed description of the model in §2. Then, in §3, we provide the equilibrium points of the system, study their stability and investigate, in detail, the effect of phenotypic differences in infection rate, virulence and mutation rate. The biological interpretations of the results are present throughout the work, however, concrete statements are found in §4. Some technical details of the bifurcation analyses are discussed in appendix A.

## 2. Mathematical model

**Figure 1.**
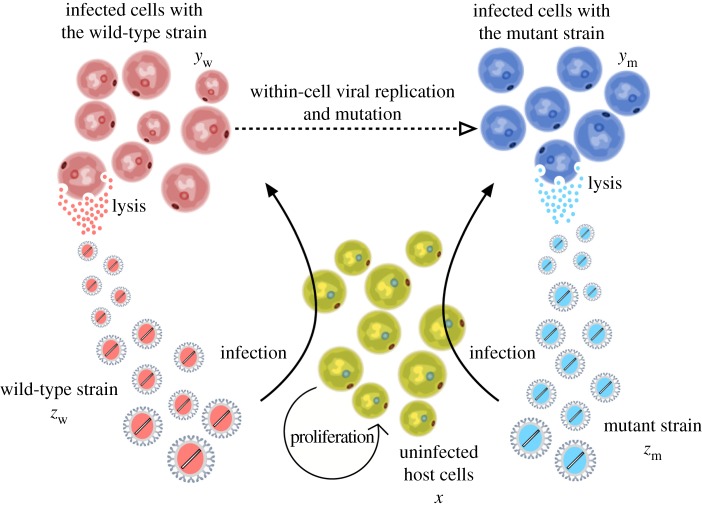
Schematic diagram of the processes included in the mathematical model for the main system investigated, which does not consider backward mutations. The system is composed of two pathogenic strains: wild-type (variable *z*_w_) and mutant (variable *z*_m_) species, that compete for the infection of healthy cells (variable *x*, green cells). Infection of healthy cells gives rise to two different populations of infected cells with wt (variable *y*_w_) and mutant (variable *y*_m_) strains, displayed by red and blue cells, respectively. Viral strains are assumed to grow and mutate (dashed lines) within the cells, being released again to the system for further infection after cell lysis.

Here we introduce the mathematical model describing the infection dynamics of wt and mutant strains. The model is based on a nonlinear system of five ordinary differential equations. The state variables of the model are: uninfected susceptible cells, *x*; two different virus strains, given by the wt (*z*_w_) and the mutant (*z*_m_) species; and two types of infected cells, one infected by the wt strain, *y*_w_, and another one infected by the mutant strains, *y*_m_ (figure 1). The time evolution of the interacting populations is described with the following model:

2.1*a* $$\dot{x}=\underset{\begin{array}{c} \mathrm{bounded}\;\mathrm{replication}\\ \mathrm{of}\;\mathrm{healthy}\;\mathrm{cells} \end{array}}{\underbrace{\beta x(1-x/K)}}\;\underset{\begin{array}{c} \mathrm{infection}\\ \mathrm{of}\;\mathrm{healthy}\;\mathrm{cells} \end{array}}{\underbrace{-\alpha_{m}z_{m}x-\alpha_{w}z_{w}x}}\;\underset{\begin{array}{c} \mathrm{decay}\;\mathrm{of}\\ \mathrm{healthy}\;\mathrm{cells} \end{array}}{\underbrace{-\delta x,}}$$

2.1*b* $${\dot{y}}_{m}=\underset{\begin{array}{c} \mathrm{growth}\;\mathrm{of}\;\mathrm{mutant}\\ \mathrm{infected}\;\mathrm{cells} \end{array}}{\underbrace{\alpha_{m}z_{m}x}}\;\overset{\begin{array}{c} \mathrm{mutation}\;\mathrm{from}\\ \mathrm{wt}\;\mathrm{to}\;\mathrm{mutant} \end{array}}{\overbrace{+\;\mu y_{w}}}\;\underset{\begin{array}{c} \mathrm{decay}\;\mathrm{of}\;\mathrm{mutant}\\ \mathrm{infected}\;\mathrm{cells} \end{array}}{\underbrace{-\gamma_{m}y_{m},}}$$

2.1*c* $${\dot{y}}_{w}=\underset{\begin{array}{c} \mathrm{growth}\;\mathrm{of}\;\mathrm{wt}\\ \mathrm{infected}\;\mathrm{cells} \end{array}}{\underbrace{\alpha_{w}z_{w}x}}\;\overset{\begin{array}{c} \mathrm{mutation}\;\mathrm{from}\\ \mathrm{wt}\;\mathrm{to}\;\mathrm{mutant} \end{array}}{\overbrace{-\;\mu y_{w}}}\;\underset{\begin{array}{c} \mathrm{decay}\;\mathrm{of}\;\mathrm{wt}\\ \mathrm{infected}\;\mathrm{cells} \end{array}}{\underbrace{-\gamma_{w}y_{w},}}$$

and

2.1*d* $${\dot{z}}_{i}=\underset{\begin{array}{c} \mathrm{lytic}\;\mathrm{release}\;\mathrm{of}\\ \mathrm{type}\;i\;\mathrm{virions} \end{array}}{\underbrace{\kappa_{i}\gamma_{i}y_{i}}}\;\overset{\begin{array}{c} \mathrm{internalization}\;\mathrm{of}\\ \mathrm{type}\;i\;\mathrm{virions}\;\mathrm{by}\;\mathrm{infection} \end{array}}{\overbrace{-\nu_{i}\alpha_{i}z_{i}x}}\;\underset{\begin{array}{c} \mathrm{degradation}\;\mathrm{of}\\ \mathrm{type}\;i\;\mathrm{virions} \end{array}}{\underbrace{-\zeta_{i}z_{i},}}\;\mathrm{with}\;\;\;i=m,\;w.$$

The terms in model (2.1) are labelled with the biological processes. The meaning of these processes as well as of the parameters is discussed in the following lines. The uninfected host cells, which are limited by the carrying capacity of their environment, *K*, proliferate and die proportionally to parameters *β* and *δ*, respectively.

As previously mentioned, the mutant and the wt strains infect the host cells at rates *α*_m_ and *α*_w_, respectively. Mathematical and statistical models of multiple infections, coinfection and superinfection have been studied in detail [49–52] and are not considered here. Acknowledging the importance of differences between lytic and lysogenic infection cycles in the production of virions, yet unlike [53–55], we consider only lytic infections, i.e. the populations of infected cells do not grow as uninfected cells do [56]. In fact, the model is based on the same assumptions of the classic Lotka–Volterra equations, which were adapted to virus dynamics by Nowak & May [57] and many others [58–62]. In the study of coexistence of viral populations, the main role of infected cells is viewed in the scope of the processes of the lytic cycle, i.e. a direct impact in change and production of free virions. Viruses replicate and produce their own type of virions via infected cells. Strains can change and mutations occur only during the process of viral replication inside an infected cell (see dashed arrow in figure 1). Therefore, cells from population *y*_w_ infected by the wt strain can mutate at rate *μ* into cells of population *y*_m_. That would increase the size of the latter population at the same rate *μ*. We note that our analyses are mainly developed considering mutations from the wt to the mutant strains. This is a standard strategy to keep the model as simple as possible, assuming that the probability of backward mutations is extremely small due to the enormous size of the sequence space. However, some results considering backward mutations will also be presented. While considering mutation in infected cells, we implicitly study the mutation of the viral genomes. It is clear that infection affects the life-span of infected cells in a nontrivial way [63]. In this study, the virulence of the strains is depicted by including new decay mechanisms for the uninfected cells. However, for simplicity, we consider death rates of infected cells to be *γ*_m_ and *γ*_w_, and as opposed to *δ* + Δ*γ*_m_ and *δ* + Δ*γ*_w_.

The absence of a mechanism for virus replication makes the multiplication of viral strains entirely dependent on the machinery of the infected cells. Therefore, the overall number of virions produced in one lytic cycle must be proportional to the virulence and the number of virions produced by a single-infected cell, the latter of which we refer to as the burst size. We average the burst sizes of viral strains and consider them as constants *κ*_m_ and *κ*_w_ for mutant- and wt-infected cells, respectively. *Au contraire*, the infection from the perspective of the virus is associated with an average number of virions spent to ensure a successful infection process. Owing to the absence of co-infection by different strains in our model, this number can be considered as the multiplicity of infection of the viral strain. We take the multiplicity of infections to be constants *ν*_m_ and *ν*_w_ for mutant and wt strains, respectively. In this model, we assume that populations are not being harvested, but do consider a decay of all the populations in the system. In the case of the virus populations, this decrease is described as an outflow or ‘death’ of free virus particles from the system. The overall ‘death’ rates of the virions in the system are ζ_w_ and ζ_m_ for wt and mutant strains, respectively.

Initial conditions for system (2.1) are non-negative values:

$$x(0)=X,\;y_{m}(0)=Y_{m},\;y_{w}(0)=Y_{w},\;z_{m}(0)=Z_{m},\;z_{w}(0)=Z_{w}.$$

### 2.1. Non-dimensionalization

The model (2.1) can be simplified by introducing dimensionless variables that are based on characteristic timescales and population sizes. The quantity *β* − *δ* describes the effective growth rate of uninfected cells and its inverse, (*β* − *δ*)^−1^, is used to define the characteristic time scale of the system. In a virus-free environment, the maximum size of the uninfected cell population is ${\tilde{x}}_{\max}=(1-\delta /\beta )K$, which is used to define the characteristic population size for both the uninfected and infected cells. The characteristic population size of the viral strains is chosen to be the product of the mutant burst size *κ*_m_ and the characteristic size of the infected cell population, ${\tilde{x}}_{\max}$. The former is required due to differences in sizes and measurement units of the viral loads and the cell populations in the system. We therefore non-dimensionalize the variables according to

2.2 $$t={\left(\beta -\delta \right)}^{-1}\bar{t},\;x={\tilde{x}}_{\max}\bar{x},\;y_{i}={\tilde{x}}_{\max}{\bar{y}}_{i},\;z_{i}={\tilde{x}}_{\max}\kappa_{m}{\bar{z}}_{i},$$

where bars are used to denote dimensionless quantities. We also introduce dimensionless parameters defined by

2.3 $${\bar{\alpha }}_{i}=\frac{\kappa_{m}K\alpha_{i}}{\beta },\;{\bar{\nu }}_{i}=\frac{\nu_{i}}{\kappa_{m}},\;\bar{\kappa }=\frac{\kappa_{w}}{\kappa_{m}},\;\bar{\mu }=\frac{\mu }{\beta -\delta },\;{\bar{p}}_{i}=\frac{\;p_{i}}{\beta -\delta },$$

where *p*_i_ (recall that i = m, w) stands for other ‘rate’ parameters, namely: *γ*_m_, *γ*_w_, ζ_m_ and ζ_w_. Notice that all non-dimensional ‘rate’ parameters, including mutation $\bar{\mu }$, are relative rates, i.e. the rate relative to the effective growth rate of uninfected cells. Meanwhile, non-dimensional parameters related to the virus populations are inevitably linked to the burst size due to the choice of ${\bar{z}}_{i}$. The non-dimensional burst size $\bar{\kappa }$ is simply the ratio of the wt and mutant burst sizes. Both multiplicity of infections are scaled with respect to the burst size of the mutant strain. The dependence of the dimensionless infection rates ${\bar{\alpha }}_{i}$ on the burst size of the mutant-type-infected cells, the carrying capacity, growth rate of uninfected cells and dimensional infection rate shows how each of these quantities affects the overall rate of infection.

Understandably, the non-dimensionalization places a restriction on the parameter values and requires *β* > *δ*. However, this restriction is biologically justified. Taking *β* > *δ*, as shown later, forces the trivial equilibrium to be unstable, thus avoiding scenarios where all populations become extinct. Upon ignoring bars for clarity purposes, we obtain the non-dimensionalized system

2.4*a* $$\dot{x}=x(1-x)-\alpha_{m}z_{m}x-\alpha_{w}z_{w}x,$$

2.4*b* $${\dot{y}}_{m}=\alpha_{m}z_{m}x+\mu y_{w}-\gamma_{m}y_{m},$$

2.4*c* $${\dot{y}}_{w}=\alpha_{w}z_{w}x-\mu y_{w}-\gamma_{w}y_{w},$$

2.4*d* $${\dot{z}}_{m}=\gamma_{m}y_{m}-\nu_{m}\alpha_{m}z_{m}x-\zeta_{m}z_{m}$$

2.4*e* $$\;\mathrm{and}\;{\dot{z}}_{w}=\kappa \gamma_{w}y_{w}-\nu_{w}\alpha_{w}z_{w}x-\zeta_{w}z_{w}.$$

## 3. Results and discussion

The dimensionless model (2.4) is now analysed to understand how the dynamics change under parameter variation. We calculate the equilibria of (2.4), conduct a linear stability analysis, and identify analytical conditions that lead to transcritical and Hopf bifurcations. We find that curves of these bifurcations can intersect at specific points in parameter space, giving rise to degenerate Bogdanov–Takens (DBT) and zero-Hopf (DZH) bifurcations. The role of DBT and DZH bifurcations is to organize the stability (or phase) diagram into regions with distinct dynamics. The numerical continuation package MATCONT [64] is used to track how the equilibria and bifurcations evolve as the infection rate, virulence, and mutation rate are varied. The MATCONT source code and data are available online [65].

### 3.1. Equilibrium points

The non-dimensional model given by equations (2.4) has four equilibria. For mathematical convenience, we define the population vector $v(t)=\{x(t),y_{m}(t),y_{w}(t),z_{m}(t),z_{w}(t)\}$. The first equilibrium is the origin

3.1 $$v_{0}\;:=\;\{x=0,\;y_{m}=0,\;y_{w}=0,\;z_{m}=0,\;z_{w}=0\}.$$

If stable, the trivial solution ***v***_0_ corresponds to the extinction of all of the populations. The second equilibrium

3.2 $$v_{1}\;:=\;\{x=1,\;y_{m}=0,\;y_{w}=0,\;z_{m}=0,\;z_{w}=0\},$$

describes, whenever stable, a virus-free state whereby only the uninfected cells persist. Owing to our choice of non-dimensionalization, the maximum population of uninfected cells is equal to one. The third equilibrium is given by

3.3 $$\begin{array}{rl} & v_{2}\;:=\;\{x=\frac{\zeta_{m}}{\alpha_{m}(1-\nu_{m})},\;\;y_{m}=\frac{(\alpha_{m}(1-\nu_{m})-\zeta_{m})\zeta_{m}}{\alpha_{m}^{2}{\left(1-\nu_{m}\right)}^{2}\gamma_{m}},\;\;y_{w}=0,\\ & \;\;z_{m}=\frac{\alpha_{m}(1-\nu_{m})-\zeta_{m}}{\alpha_{m}^{2}(1-\nu_{m})},\;\;z_{w}=0\}, \end{array}$$

and, if stable, corresponds to the persistence of uninfected cells and the mutant strain of the virus. There is no wt strain of the virus and the viral population is only composed of mutant genotypes. In order for the wt-free state ***v***_2_ to be biologically meaningful, the condition *ν*_m_ < 1 must hold. This condition corresponds to the burst size of the mutant virus being greater than its multiplicity of infection. Throughout the remainder of the paper, it will be assumed that *ν*_m_ < 1. Finally, the fourth equilibrium point is given by

3.4 $$\begin{array}{rl} v_{3} & :=\{x=\frac{\zeta_{w}(\gamma_{w}+\mu )}{A\alpha_{w}},\;y_{m}=\frac{B\mu \zeta_{w}(C+\alpha_{m}\gamma_{w}\zeta_{w})}{A^{2}\;C\alpha_{w}^{2}\gamma_{m}},\;y_{w}=\frac{B\zeta_{w}(C-\alpha_{m}\mu \zeta_{w})}{A^{2}C\alpha_{w}^{2}},\\ & \;z_{m}=\frac{B\mu \zeta_{w}}{AC\alpha_{w}},\;z_{w}=\frac{B(C-\alpha_{m}\mu \zeta_{w})}{AC\alpha_{w}^{2}}\}, \end{array}$$

where

3.5 $$\begin{array}{rl} A & =\gamma_{w}\kappa -(\mu +\gamma_{w})\nu_{w},\\ B & =-((\nu_{w}-\kappa )\alpha_{w}+\zeta_{w})\gamma_{w}-\mu (\alpha_{w}\nu_{w}+\zeta_{w})\\ \;\mathrm{and}\;\;C & =\alpha_{m}\zeta_{w}(\gamma_{w}(1-\nu_{m})-\mu \nu_{m})-\alpha_{w}\;\zeta_{m}(\gamma_{w}(\kappa -\nu_{w})-\mu \nu_{w}). \end{array}\}$$

This equilibrium point involves, whenever stable, a state of coexistence for the wt and mutant strains. Similar to the wt-free state ***v***_2_, the coexistence state ***v***_3_ can only be biologically meaningful if the burst size of wt strain satisfies *κ* > *ν*_w_(1 + *μ*/*γ*_w_). This inequality is a generalization of that derived for the mutant virus (*ν*_m_ < 1) which accounts for mutation. The singularity that occurs in the coexistence state ***v***_3_ when *C* = 0 leads to difficulties when using numerical methods to track how this equilibrium evolves under parameter variation. The assumption of uni-directional mutation prevents the existence of an equilibrium point that is analogous to ***v***_2_ whereby only the wt virus exists.

### 3.2. Linear stability analysis and bifurcations

A linear stability analysis is carried out to determine the behaviour of the system close to the equilibrium points. The Jacobian matrix for equations (2.4) is given by

$$J=\left[\begin{array}{ccccc} -\alpha_{m}z_{m}-\alpha_{w}z_{w}-2x+1 & 0 & 0 & -\alpha_{w}x & -\alpha_{m}x\\ \alpha_{w}z_{w} & -\gamma_{w}-\mu & 0 & \alpha_{w}x & 0\\ \alpha_{m}z_{m} & \mu & -\gamma_{m} & 0 & \alpha_{m}x\\ -\alpha_{w}\nu_{w}z_{w} & \kappa \gamma_{w} & 0 & -\alpha_{w}\nu_{w}x-\zeta_{w} & 0\\ -\alpha_{m}\nu_{m}z_{m} & 0 & \gamma_{m} & 0 & -\alpha_{m}\nu_{m}x-\zeta_{m} \end{array}\right].$$

Straightforward calculations for the trivial solution ***v***_0_ yield eigenvalues of the Jacobian given by

3.6 $$\Lambda_{0}={\left(1,\;-\zeta_{w},\;-\zeta_{m},\;-\gamma_{m},\;-\gamma_{w}-\mu \right)}^{T}.$$

Thus, for all parameter values, the trivial solution ***v***_0_, as mentioned before, is always unstable, i.e. it is a saddle point with a one-dimensional unstable manifold.

Although it is possible to find analytical expressions for the eigenvalues of the Jacobian evaluated at ***v***_1_, they are sufficiently complicated that little insight is gained from analysing them directly. In order to conduct a stability analysis for ***v***_1_, it is more useful to consider the characteristic polynomial for the eigenvalues λ. The polynomial $\det(J(v_{1})-\lambda I)=0$ can be regrouped into the form of three factors $P_{1}(\lambda )\cdot P_{2}(\lambda )\cdot P_{3}(\lambda )=0$, where

3.7*a* $$P_{1}(\lambda )=\lambda +1,$$

3.7*b* $$P_{2}(\lambda )=\lambda^{2}+(\alpha_{w}\nu_{w}+\gamma_{w}+\mu +\zeta_{w})\lambda -\alpha_{w}(\gamma_{w}(\kappa -\nu_{w})-\mu \nu_{w})+\zeta_{w}(\gamma_{w}+\mu )$$

3.7*c* $$\;\mathrm{and}\;P_{3}(\lambda )=\lambda^{2}+(\alpha_{m}\nu_{m}+\gamma_{m}+\zeta_{m})\lambda +\alpha_{m}\gamma_{m}(\nu_{m}-1)+\gamma_{m}\zeta_{m}.$$

The first factor, *P*_1_, provides a constant eigenvalue, λ = −1, which reserves the possibility for stability of ***v***_1_. Although the next two factors *P*_2_ and *P*_3_ do not provide obvious eigenvalues, they enable the identification of critical parameter groups for which ***v***_1_ undergoes a bifurcation.

Based on our knowledge of the bifurcations that can occur in models similar to (2.4), we may expect to find transcritical and Hopf bifurcations. For a varying parameter (or set of parameters), the onset of a Hopf bifurcation leads to the creation of periodic orbits (POs) after the change of stability of the equilibrium point. Furthermore, Hopf bifurcations are characterized by the Jacobian matrix having a single pair of complex conjugate eigenvalues with zero real part. An analysis of the factors *P*_2_ and *P*_3_ given by (3.7b,c) reveals that ***v***_1_ cannot undergo a Hopf bifurcation. This is because the coefficients of the linear terms are strictly positive, thus preventing the eigenvalues from ever being purely imaginary.

Transcritical bifurcations occur when two equilibria collide non-destructively, exchanging their stability and resulting in the Jacobian matrix having a single eigenvalue that is equal to zero. Since ***v***_1_ exists for all parameter combinations, transcritical bifurcations can be straightforwardly detected by forcing λ to be zero in *P*_2_ and *P*_3_. By solving *P*_2_(λ = 0) = 0, we find $R_{0}^{w}=1$, where

3.8 $$R_{0}^{w}:=\frac{\alpha_{w}}{\zeta_{w}}\left(\frac{\kappa }{1+\mu \gamma_{w}^{-1}}-\nu_{w}\right).$$

Similarly, solving *P*_3_(λ = 0) = 0 results in $R_{0}^{m}=1$, where

3.9 $$R_{0}^{m}:=\frac{\alpha_{m}}{\zeta_{m}}(1-\nu_{m}).$$

It will be shown below that $R_{0}^{w}$ and $R_{0}^{m}$ are the basic reproductive numbers for the wt and mutant strains, respectively. When $R_{0}^{w}=1$, the virus-free state ***v***_1_ and the coexistence state ***v***_3_ intersect. By expanding ***v***_3_ around $R_{0}^{w}=1$, we find that it has negative components when $R_{0}^{w}<1$ and thus lies outside of the biologically meaningful phase space. However, all of the components of ***v***_3_ become positive when $R_{0}^{w}>1$. Likewise, expanding the equilibrium ***v***_2_ around $R_{0}^{m}=1$ shows that this condition corresponds to points where the virus-free state ***v***_1_ and wt-free state ***v***_2_ intersect. For $R_{0}^{m}<1$, some components of ***v***_2_ are negative; for $R_{0}^{m}>1$, all components are positive. In the case when both $R_{0}^{w}=R_{0}^{m}=1$ hold, there is a triple intersection of ***v***_1_, ***v***_2_ and ***v***_3_. Furthermore, the Jacobian has a double zero eigenvalue at this point, indicating the onset of a non-degenerate or degenerate Bogdanov–Takens bifurcation. As will be shown in §3.1, the Bogdanov–Takens bifurcations in this model are of degenerate type. More generally, we find that ***v***_2_ and ***v***_3_ intersect when $R_{0}^{m}=R_{0}^{w}$, that is, when

3.10 $$\frac{\alpha_{w}}{\zeta_{w}}\left(\frac{\kappa }{1+\mu \gamma_{w}^{-1}}-\nu_{w}\right)=\frac{\alpha_{m}}{\zeta_{m}}(1-\nu_{m}).$$

Thus, there are three curves of transcritical bifurcations defined by $R_{0}^{w}=1$, $R_{0}^{m}=1$ and $R_{0}^{m}=R_{0}^{w}$, all of which simultaneously intersect at the DBT point.

**Figure 2.**
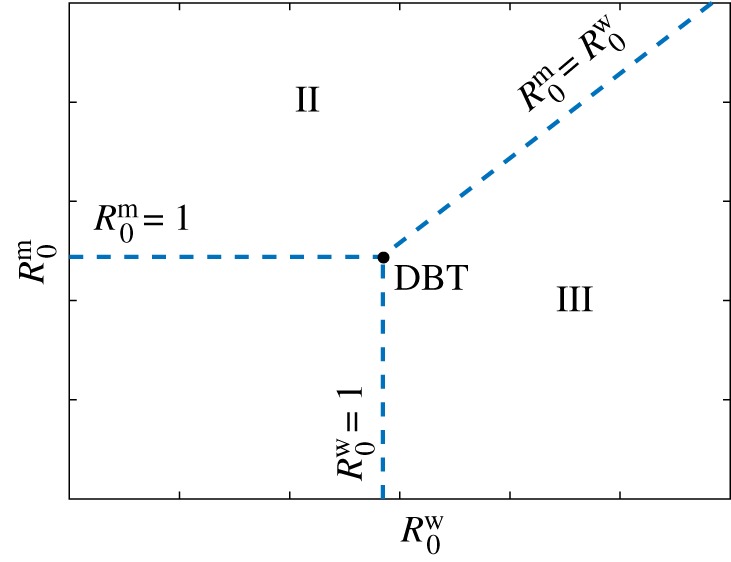
Co-dimension two bifurcation diagram displaying the local stability near the degenerate Bogdanov–Takens (DBT) bifurcation in terms of the basic reproduction numbers defined in (3.8) and (3.9). Three regions with different qualitative behaviours are identified. Region I: virus-free state without infected cells (***v***_1_ stable); Region II: wt-free state (***v***_2_ stable); and Region III: coexistence of all strains (***v***_3_ stable).

The dynamics of the system near the DBT bifurcation can be determined by expanding the equilibria and their eigenvalues around $R_{0}^{w}=1$ and $R_{0}^{m}=1$. The eigenvalues of all of the equilibria are real, ruling out the possibility of a branch of Hopf bifurcations emanating from the DBT point, which is a generic feature of non-degenerate Bogdanov–Takens bifurcations [66]. When $R_{0}^{w}<1$ and $R_{0}^{m}<1$, the virus-free state ***v***_1_ is locally asymptotically stable and the wt-free state ***v***_2_ and the coexistence state ***v***_3_ are unstable, meaning that the virus-free state is achieved. For $R_{0}^{m}>1$ and $R_{0}^{w}<1$, the virus-free and wt-free state exchange stability, with ***v***_1_ becoming unstable and ***v***_2_ locally asymptotically stable, with ***v***_3_ remaining unstable. Similarly, for $R_{0}^{w}>1$ and $R_{0}^{m}<1$, the virus-free and coexistence state exchange stability: ***v***_1_ becomes unstable, ***v***_3_ becomes locally asymptotically stable, and ***v***_2_ remains unstable. The stability in the region $R_{0}^{m}>1$ and $R_{0}^{w}>1$ is governed by the transcritical bifurcation occurring along the curve $R_{0}^{m}=R_{0}^{w}$, which leads to an exchange of stability between wt-free and coexistence states, ***v***_2_ and ***v***_3_. The transcritical bifurcations that occur for $R_{0}^{m}=1$ with $R_{0}^{w}>1$, $R_{0}^{w}=1$ with $R_{0}^{m}>1$ and $R_{0}^{m}=R_{0}^{w}$ with $R_{0}^{w}<1$ do not result in an exchange of stability. Thus, the local stability diagram near the DBT bifurcation can be drawn as in figure 2. There are three distinct regions, denoted by I, II and III, where ***v***_1_, ***v***_2_ and ***v***_3_ are local attractors, respectively.

Based on these results, we can interpret the quantities $R_{0}^{m}$ and $R_{0}^{w}$ as basic reproduction numbers for the mutant and wt viral strains, respectively. In general, for a strain to persist, its basic reproduction number has to be strictly greater than one. Besides, the value of a basic reproduction number is proportional to the number of new infections arising in a following generation of virions from an infected cell. Identifying basic reproduction numbers enables different models of population dynamics to be compared in a consistent manner. However, as it is not possible to write the dimensionless model (2.4) solely in terms of the basic reproduction numbers $R_{0}^{m}$ and $R_{0}^{w}$, we will consider how the dynamics change under the variation of specific individual parameters that are of biological interest.

To examine the stability of the wt-free state ***v***_2_, we obtain the eigenvalues from the characteristic polynomial computed from $\det(J(v_{2})-\lambda I)=0$. This equation can be factorized and rewritten as $Q_{2}(\lambda )\cdot Q_{3}(\lambda )=0$, where *Q*_2_ and *Q*_3_ are quadratic and cubic polynomials in λ, respectively. The exact form of *Q*_2_ is not required here. We write *Q*_3_(λ) = *a*λ^3^ + *b*λ^2^ + *c*λ + *d* = 0, where

3.11 $$\begin{array}{ll} & a=\alpha_{\text{m}}{\left(1-\nu_{\text{m}}\right)}^{2},\;\\ & b=(1-\nu_{\text{m}})(\alpha_{\text{m}}\gamma_{\text{m}}(1-\nu_{\text{m}})+\zeta_{\text{m}}(\alpha_{\text{m}}+1)),\\ & c=\zeta_{\text{m}}(\nu_{\text{m}}^{2}\alpha_{\text{m}}+(\zeta_{\text{m}}-\alpha_{\text{m}}-\gamma_{\text{m}})\nu_{\text{m}}+\zeta_{\text{m}}+\gamma_{\text{m}})\\ \text{and} & d=(1-\nu_{\text{m}})\zeta_{\text{m}}\gamma_{\text{m}}(\alpha_{\text{m}}(1-\nu_{\text{m}})-\zeta_{\text{m}}).\; \end{array}\}$$

Following the analysis scheme discussed earlier, to obtain conditions for Hopf bifurcations of ***v***_2_, we search for a purely imaginary pair of eigenvalues. Setting *Q*_3_(λ = ±*iω*) = 0, we obtain the critical condition for a Hopf bifurcation, *ad* = *bc*, with $\omega =\sqrt{d/b}$ being the angular frequency of the emerging POs. Interestingly, for this Hopf bifurcation, there is no dependence on parameters associated with the wt strain of virus. For all biologically meaningful solutions of *ad* = *bc*, the mutant strain of virus has the potential to gain periodic behaviour through the creation of a stable PO. The other factor, *Q*_2_(λ), provides no possibility for a Hopf bifurcation due to a strictly positive linear coefficient in the quadratic polynomial.

Transcritical bifurcations of ***v***_2_ may occur if *Q*_2_(λ = 0) = 0 or *Q*_3_(λ = 0) = 0. First, setting *Q*_3_(λ = 0) = 0 yields *d* = 0, which is equivalent to $R_{0}^{m}=1$ and corresponds to an intersection of ***v***_1_ and ***v***_2_. Second, from *Q*_2_(λ = 0) = 0, we obtain an expression which matches with (3.10), that is, the parameter combination yielding an intersection of ***v***_2_ and ***v***_3_.

As will be shown in §3.3, it is possible to simultaneously satisfy *Q*_3_(λ = ±*iω*) = 0 and *Q*_2_(λ = 0) = 0, implying that the Jacobian matrix at $v_{2}=v_{3}$ has a pair of purely imaginary complex conjugate eigenvalues and a zero eigenvalue. This corresponds to the onset of a degenerate zero-Hopf (DZH) bifurcation. Like the DBT bifurcation, the DZH bifurcation will be shown to have an unusual structure that does not coincide with any of the standard normal forms (this point is further discussed in appendix A). In particular, our analysis reveals that a curve of global transcritical bifurcations of POs (TPO bifurcation) emanates from the DZH point rather than a curve of torus bifurcations [66]. A TPO bifurcation occurs when an unstable PO and a stable PO collide with each other in a non-destructive way (differently from what would happen in a saddle-node bifurcation of POs, with destruction of POs), and subsequently exchange stability.

The detailed stability and bifurcation analysis of ***v***_2_, ***v***_3_, as well as the DZH point, will be performed numerically. In particular, we will construct one- and two-dimensional bifurcation diagrams using specific pairs of parameters.

### 3.3. Phenotypic differences in infection rates

We first examine the influence of the infection rates *α*_m_ and *α*_w_ on the dynamics. The values of the other parameters are set to

3.12 $$\mu =0.1,\;\kappa =1,\;\gamma_{m}=\gamma_{w}=0.25,\;\nu_{m}=\nu_{w}=0.5,\;\zeta_{m}=\zeta_{w}=0.2222.$$

**Figure 3.**
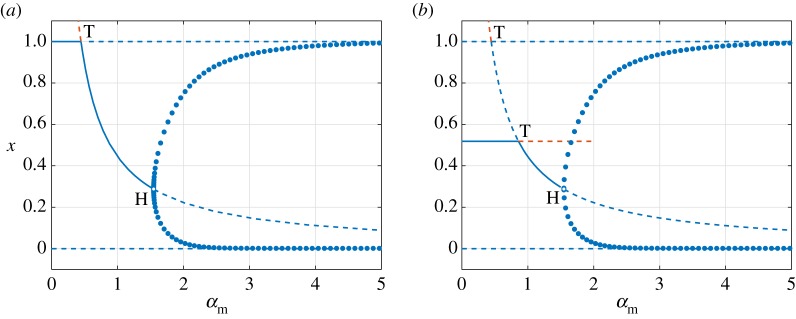
One-dimensional bifurcation diagrams showing the dependence of the equilibrium population of healthy cells *x* on the infection rate of mutant-type virus *α*_m_. We set (*a*) *α*_w_ = 0.5 and $R_{0}^{w}=0.48$ and (*b*) *α*_w_ = 2 and $R_{0}^{w}=1.93$. Solid and dashed curves illustrate, respectively, stable and unstable equilibria. Filled markers stand for maxima and minima of a stable periodic orbit (PO). The letters T and H denote points of transcritical and Hopf bifurcations of equilibria, respectively. Blue denotes biologically relevant solutions (positive variables) while orange describes solutions with negative components. The minimum values of the POs are above zero as *α*_m_ is increased after the Hopf bifurcation. All the other parameter values are given in (3.12).

We begin our investigation by constructing one-dimensional bifurcation diagrams using *α*_m_ as the bifurcation parameter and fixing *α*_w_. We first consider two values of the wt strain infections rate given by *α*_w_ = 0.5 and *α*_w_ = 2, corresponding to basic reproduction numbers $R_{0}^{w}=0.48$ and $R_{0}^{w}=1.93$, respectively. These values of *α*_w_ therefore lie on opposite sides of the curve of transcritical bifurcations involving ***v***_1_ and ***v***_3_ defined by $R_{0}^{w}=1$. The resulting bifurcation diagrams are shown in figure 3. Solid and dashed lines represent stable and unstable equilibria, respectively. Solid circles represent maxima and minima of stable POs. Blue and orange lines denote equilibria that exist in biologically meaningful and non-meaningful phase space, respectively. The parametric dependence of the population of uninfected cell population *x* is used as this is the only component that differs between all four equilibria.

Figure 3*a* shows, for the case *α*_w_ = 0.5, two critical values of the mutant-virus infection rate *α*_m_ that lead to qualitative changes in the dynamics of the system. The point *α*_m_ = 0.44 corresponds to $R_{0}^{m}=1$, and marks the position of the transcritical bifurcation involving the virus-free state ***v***_1_ and the wt-free state ***v***_2_. As predicted by the linear stability analysis in §3.2, the wt-free state undergoes a supercritical Hopf bifurcation at *α*_m_ = 1.55. The two critical values of *α*_m_ divide the parameter space into three distinct regions. For *α*_m_ < 0.44, the virus-free state ***v***_1_ is locally asymptotically stable, corresponding to Region I in figure 2. Similarly, for 0.44 < *α*_m_ < 1.55, the wt-free state ***v***_2_ is stable, corresponding to Region II of figure 2. Finally, for *α*_m_ > 1.55, there is a new region, termed Region IV, where stable POs exist about the equilibrium ***v***_2_, which is unstable. These POs describe oscillatory populations of uninfected cells and mutant-type virus with extinct populations of the wt virus.

When the infection rate of wt virus is increased to *α*_w_ = 2, the virus-free and coexistence states undergo a transcritical bifurcation. Consequently, the bifurcation diagram in figure 3*b* shows that for mutant-type infection rates given by *α*_m_ < 0.86, the coexistence state ***v***_3_ is stable whereas the virus-free state ***v***_1_ is unstable. This implies that Region I (***v***_1_ stable) is replaced by Region III (***v***_3_ stable). Although ***v***_1_ and ***v***_2_ undergo a transcritical bifurcation at *α*_m_ = 0.44, there is no exchange of stability. Instead, it is ***v***_2_ and ***v***_3_ which exchange stability through a transcritical bifurcation at *α*_m_ = 0.86, corresponding to the case of equal basic reproduction numbers, $R_{0}^{m}=R_{0}^{w}=1.93$. Both Regions II and IV persist for *α*_w_ = 2, with the supercritical Hopf bifurcation occurring at *α*_m_ = 1.55, the same location as in figure 3*a*.

The one-dimensional bifurcation diagrams shown in figure 3 can be rebuilt using the infection rate of the wt virus *α*_w_ as the bifurcation parameter for fixed values of *α*_m_ that lie on opposite sides of $R_{0}^{m}=1$. However, the resulting diagrams are qualitatively similar to those in figure 3, with the exception that the wt-free state ***v***_2_ is switched with the coexistence state ***v***_3_ and *vice versa*. The supercritical Hopf bifurcation now occurs from ***v***_3_ and thus gives rise to Region V, characterized by the periodic coexistence of the viral populations.

**Figure 4.**
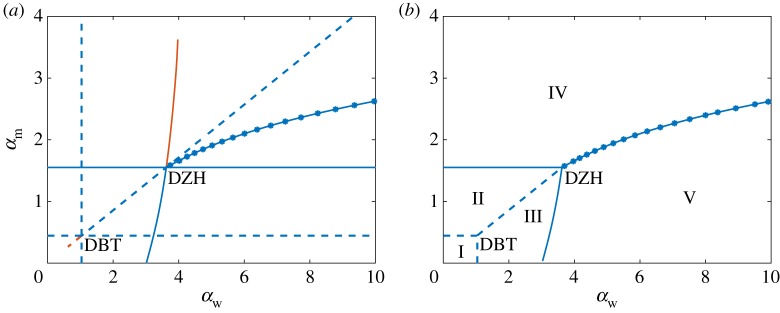
(*a*) Two-dimensional bifurcation diagram, illustrating the effect of infection rates on the dynamics of the system. Blue denotes biologically meaningful solutions (positive values for all components). Orange describes solutions with negative components, which are not biologically meaningful. The abbreviations DBT and DZH mark the locations of degenerate Bogdanov–Takens and degenerate zero-Hopf bifurcations, respectively. (*b*) Two-dimensional stability diagram with the biologically meaningless bifurcations removed, showing five regions of distinct dynamics. Solid, dashed and starred lines represent curves of Hopf bifurcations, transcritical bifurcations of fixed points and transcritical bifurcations of periodic orbits (POs). Region I: stability of virus-free solution; Region II: stability of wt-free state; Region III: stability of coexistence state; Region IV: stable PO around the wt-free state; Region V: stable PO around the coexistence state. In both plots, the parameter values are given by (3.12).

The five regions identified from the bifurcation analysis can be conveniently visualized by constructing a two-dimensional bifurcation diagram where both infection rates *α*_m_ and *α*_w_ are continuously varied. In this diagram, displayed in figure 4*a*, the locations of the bifurcations that separate the five regions are traced out as the infection rates vary. Not all of the bifurcations shown in figure 4*a* lead to a biologically meaningful change in the dynamics, i.e. they do not represent a boundary between two distinct regions. Thus, in figure 4*b* we represent the two-dimensional diagram with only the biologically meaningful bifurcations shown.

Figure 4*a* reveals a complicated bifurcation scenario that is largely centred about DBT and DZH bifurcations occurring at *α*_w_ = 1.04, *α*_m_ = 0.44 and *α*_w_ = 3.62, *α*_m_ = 1.55, respectively. The DBT bifurcation lies at the intersection of three curves of transcritical bifurcations satisfying $R_{0}^{w}=1$, $R_{0}^{m}=1$ and $R_{0}^{m}=R^{w}$, which may be written, respectively, in terms of the infection rates as

3.13 $$\alpha_{w}=\frac{\zeta_{w}}{\kappa {\left(1+\mu \gamma_{w}^{-1}\right)}^{-1}-\nu_{w}},\;\alpha_{m}=\frac{\zeta_{m}}{1-\nu_{m}},\;\frac{\alpha_{w}}{\alpha_{m}}=\frac{\zeta_{w}}{\zeta_{m}}\frac{1-\nu_{m}}{\kappa {\left(1+\mu \gamma_{w}^{-1}\right)}^{-1}-\nu_{w}}.$$

**Figure 5.**
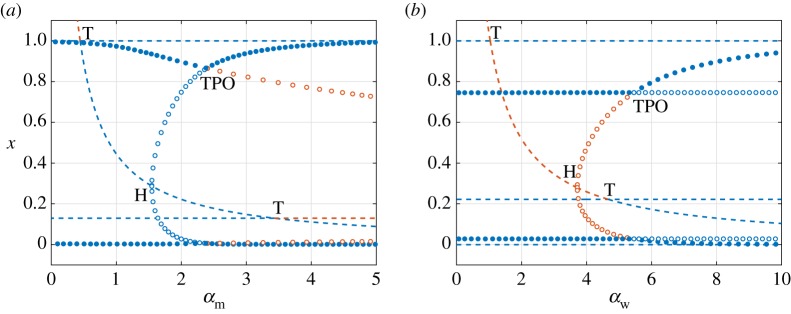
One-dimensional bifurcation diagrams for fixed values of (*a*) *α*_w_ = 8 and (*b*) *α*_m_ = 2, with only the *x*-component of equilibria (population of healthy cells) plotted for clarity. Dashed curves illustrate unstable equilibria. Filled and open markers stand for stable and unstable periodic orbits (POs), respectively. Only the maxima and minima of each PO is shown. Blue denotes biologically meaningful solutions and POs (strictly positive values for all components). Orange describes biologically meaningless solutions with negative components. Parameter values are given in (3.12). The letter T represents a transcritical bifurcation of equilibria, H a Hopf bifurcation, and TPO a transcritical bifurcation of POs.

The bifurcation structure around the DBT point is identical to that predicted from the local stability analysis. Thus, we can eliminate from figure 4*a* the biologically irrelevant curves of transcritical bifurcations emerging from the DBT point in order to obtain the boundaries between Regions I, II and III shown in figure 4*b*. The DZH bifurcation occurs at the simultaneous intersection of two curves of Hopf bifurcations associated with ***v***_2_ and ***v***_3_ and the curve of transcritical bifurcations involving ***v***_2_ and ***v***_3_. As predicted from linear stability analysis, the Hopf bifurcation curve associated with the wt-free state ***v***_2_ does not depend on the infection rate of the wt virus *α*_w_ and thus appears as a straight line given by *α*_m_ = 1.55. Emanating from the DZH point is a curve of TPO bifurcations. To better understand the dynamics that occur near the DZH point, one-dimensional bifurcation diagrams are obtained by setting *α*_w_ = 8 and treating *α*_m_ as a bifurcation parameter and then setting *α*_m_ = 2 and treating *α*_w_ as the bifurcation parameter. The resulting diagrams are shown in figure 5, with open circles denoting unstable POs.

Figure 5*a* shows that as *α*_m_ is increased from zero when *α*_w_ = 8, the wt-free state ***v***_2_ first undergoes a transcritical bifurcation with the virus-free state ***v***_1_, then a subcritical Hopf bifurcation, and finally a transcritical bifurcation with the coexistent state ***v***_3_. None of these bifurcations change the stability of the equilibria. Thus, the associated curves of transcritical and Hopf bifurcations in figure 4*a* do not represent boundaries between the five characteristic regions and are not shown in figure 4*b*. For these infection rates, all of the equilibria are unstable and the dynamics are therefore determined by the stability of POs. For sufficiently small values of *α*_m_, the system is in Region V, and there is a stable PO around the coexistence state ***v***_3_. This PO is created by the Hopf bifurcation that defines the boundary between Regions III and V shown in figure 4*b*. As *α*_m_ is increased, the unstable POs around the wt-free state ***v***_2_ that emerge from the subcritical Hopf bifurcation grow in size. Eventually, this unstable PO collides and exchanges stability with the stable PO around ***v***_3_ through a TPO bifurcation at *α*_m_ = 2.3972. During the TPO bifurcation, the stable and biologically meaningful PO around ***v***_3_ becomes unstable and some components enter negative phase space. Afterwards, the PO around the wt-free state is stable and the system is in Region IV.

The one-dimensional bifurcation diagram created with *α*_m_ = 2 and shown in figure 5*b* shares the same qualitative features as figure 5*a*, with ***v***_2_ and its POs exchanging roles with ***v***_3_ and its POs. None of the bifurcation of equilibria have biological significance. The main difference in this case is that the subcritical Hopf bifurcation and the POs it creates exist outside the biologically meaningful phase space. As these POs are involved in a biologically meaningful TPO bifurcation, this figure demonstrates how it can still be useful to understand and resolve features that lie outside of the biologically meaningful space.

Upon removing the biologically redundant bifurcation curves from figure 4*a*, the stability diagram in figure 4*b* is obtained. The transcritical bifurcations at the boundaries between Regions I and II, and I and III, capture the transition from virus-free to virus-persistent states that tend to a stable equilibrium. Supercritical Hopf bifurcations separate Regions II from IV and III from V, and mark the transition between stationary and periodic population dynamics. The curve of TPO bifurcations separates Regions IV and V, both of which are characterized by periodic viral populations, in the same way that a curve of transcritical bifurcations separates Regions II and III, which describe stationary viral populations. Thus, despite the system exhibiting a range of complex dynamics, they can be elegantly classified and organized in terms of the regions shown in figure 4*b*.

Overall, in figure 4*b*, we notice quite a large area of coexistence, Regions III and V. In population genetics, this corresponds to the most simple case of the emergence and persistence of a polymorphism in a population and maintenance of biodiversity. An example of an experiment that illustrates a simplified competition was conducted for *Escherichia coli* in [67]. These experiments focused on studying the behaviour of a newly emerging mutant in a population of a few strains of bacteria that compete with each other in a spatially homogeneous environment for the same type of nutrients. The results of the competition experiments demonstrate that in the vast majority of cases, the competitors stably coexist in steady or periodic states, which align with the outcome of our theoretical model. When even a slight change is possible in the genome, new mutations will appear. However, if all the other characteristics of an initial and a mutant population are the same, then, for both populations to persist, the initial population must have larger fitness than a mutant population. Otherwise, the initial population will be out-competed because of the constant ‘leak’ into the mutant population. A more detailed analysis of the impact of mutation rates in the dynamics can be found in §3.5.

### 3.4. Phenotypic differences in virulence

We now study the effect of the strains’ virulence (parametrized by *γ*_w_ and *γ*_m_) on the dynamics of the system by building bifurcation diagrams. The infection rates are chosen to be from the different regions of the stability diagram shown in figure 4*b*, which are based on the reference values *γ*_m_ = *γ*_w_ = 0.25. The other parameters are fixed to

3.14 $$\mu =0.1,\;\kappa =1,\;\nu_{m}=\nu_{w}=0.5,\;\zeta_{m}=\zeta_{w}=0.2222.$$

We focus on Regions I, III and IV of figure 4*b* as a starting point, covering all the other regions from there.

We begin with the case where the infection rates are chosen to coincide with Region I at the virulence reference values. These infection rates therefore correspond to basic reproduction numbers that are less than one. From the definition of $R_{0}^{m}$ given by (3.9), we see that the basic reproduction number of the mutant is independent of the virulence. Thus, changes in *γ*_m_ or *γ*_w_ cannot increase $R_{0}^{m}$ beyond one. Therefore, Regions II and IV, where the mutant-type virus persists at the expense of the wt virus becoming extinct, cannot be entered. The basic reproduction number for the wt virus given by (3.8) is an increasing function of the virulence of the wt virus. In the limit of very large virulence, *γ*_w_ → ∞, we find that $R_{0}^{w}\to \alpha_{w}(\kappa -\nu_{w})/\zeta_{w}$. Thus, if the infection rate *α*_w_ is so small that the limit of $R_{0}^{w}$ is less than one, then it will not be possible to leave Region I and both strains of the virus will always become extinct. However, if *α*_w_ is sufficiently large and the basic reproduction number increases beyond one, then a change in dynamics will be observed as *γ*_w_ is increased. By solving $R_{0}^{w}=1$, a critical value of the virulence of the wt virus is obtained

3.15 $$\gamma_{w}^{\mathrm{crit}}=\frac{\mu }{\kappa {\left(\zeta_{w}\alpha_{w}^{-1}+\nu_{w}\right)}^{-1}-1}.$$

The above equation is the condition for a transcritical bifurcation between the virus-free state ***v***_1_ and the coexistence state ***v***_3_ and defines the boundary between Regions I and III. Thus, only for values of $\gamma_{w}>\gamma_{w}^{\mathrm{crit}}$ will the virus persist in the system for this choice of infection rates.

**Figure 6.**
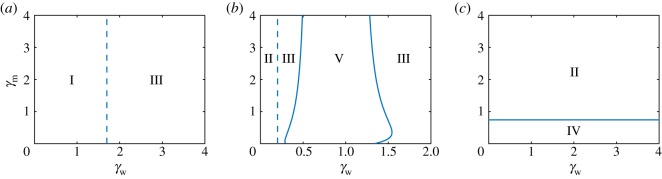
Two-dimensional bifurcation diagrams for *γ*_w_ versus *γ*_m_ at different sets of *α*_i_, with *i* = *m*, *w*. Parameter values are given by (3.14) and (*a*) *α*_w_ = 0.5, *α*_m_ = 0.1; (*b*) *α*_w_ = 3, *α*_m_ = 1; and (*c*) *α*_w_ = 0.5, *α*_m_ = 2. Dashed and solid lines correspond to transcritical and Hopf bifurcations, respectively. Region I: stability of trivial solution; Region II: stability of wt-free state; Region III: stability of coexistence state; Region IV: stable periodic orbit (PO) around the wt-free state; Region V: stable PO around the coexistence state.

The stability diagram for *α*_w_ = 0.5 and *α*_m_ = 0.1 has been numerically computed and is shown in figure 6*a*. These values of the infection rate correspond to Region I at the reference values of the virulence (figure 4*b*). As predicted, Regions II and IV are absent from the stability diagram. However, Region V is also missing. Thus, for this choice of infection rates, only Regions I and III can be entered by changing the values of the virulence. Regions I and III are separated by a straight dash line given by the critical condition (3.15).

We now consider infection rates given by *α*_w_ = 3, *α*_m_ = 1, which correspond to Region III at the reference virulence (figure 4*b*). The resulting stability diagram in terms of the virulence is shown in figure 6*b*. The choice of *α*_m_ = 1 along with the values of the parameters in (3.14) leads to $R_{0}^{m}=2.25$. Thus, variations in the virulence cannot bring the system to Region I and at least one type of virus will always persist. A transition between Regions II and III can occur via the transcritical bifurcation between the wt-free and coexistence states ***v***_2_ and ***v***_3_. The critical condition for this transcritical bifurcation is given by equation (3.10), and depends only on the virulence of the wt viral strain. Thus, the transition between Regions II and III appears as the vertical dashed line in figure 6*b*. Interestingly, this figure shows that as the virulence of the wt virus is increased, the system transitions from Region III to Region V and then back to Region III. These transitions occur via supercritical Hopf bifurcations, denoted by solid lines. This scenario corresponds to the so-called bubble bifurcation (found in other epidemiological systems, e.g. [68]), in which the system is in a stationary state, then enters into an oscillating one, and finally goes back to a stationary state as the control parameter is changed.

To understand why Region IV does not appear in the stability diagrams on figure 6*a*,*b*, we first recall that Region IV is separated from Region II by a Hopf bifurcation curve of the wt-free state ***v***_2_. This Hopf curve has been calculated analytically from the equality *ad* = *bc*, where *a*, *b*, *c* and *d* are given in (3.11), and is a quadratic expression for *γ*_m_ independent of *γ*_w_ and *α*_w_. Solving *ad* = *bc* to find $\gamma_{m}^{\mathrm{crit}}$ (not shown due to complexity of the expression), at the values of infection rates chosen for figure 6*a*,*b*, we notice they are outside of the positive parameters space. In fact, only for values of the mutant-type infection rate that satisfy

3.16 $$\alpha_{m}>\frac{\zeta_{m}}{1-\nu_{m}}\left(1+\frac{1}{\nu_{m}}\right),$$

equivalent to *α*_m_ > 1.3332 for the chosen parameters, does the Hopf curve of ***v***_2_ appear on a *γ*_w_ versus *γ*_m_ stability diagram (figure 6*c*). Greater values of *α*_m_ lead to increases in the area of Region IV under Region II.

The stability diagrams of figure 6 indicate the impact of virulence on the complexity of the dynamics. Clearly, the virulence of the mutant strain does not affect the survival of the wt strain. That is, increases in *γ*_m_ do not lead to an appearance or disappearance of the wt strain. However, the survival of the mutant strain does not appear to depend on *γ*_m_ either. Importantly, the virulence of the wt strain, *γ*_w_, strongly controls the dynamics and the persistence of the wt strain. As can be seen for small values of *γ*_w_, this strain can become extinct as shown in figure 6*b*. In other words, even for a superior infection rate of the wt viral strain, there is a threshold of *γ*_w_ that must be surpassed in order for this strain to exist. For inferior values that are below this threshold, the survival of the wt strain is impossible because the rate of wt virion production, i.e. the death of rate infected cells, is insufficient. The qualitative behaviour of solutions, i.e. whether the populations of wt strains undergo oscillations or stabilize to a certain value, depends on the virulence in a non-trivial way. As shown in figure 6*b*, there is an interval of values for *γ*_w_ for which the system has a stable PO governing the coexistence of strains, marked as Region V. Outside of this interval, the populations reach stable stationary states.

### 3.5. The effect of heterogeneity in mutation rates

In the previous analyses, the mutation rate has been fixed to *μ* = 0.1. We now consider the effect of *μ* on the dynamics. This is a key parameter that has been largely investigated within the framework of the error threshold [69,70] and lethal mutagenesis [71] in quasi-species theory. In general, the coexistence of two strains requires the basic reproduction number of the wt virus to be greater than one, $R_{0}^{w}>1$, which can be interpreted as a condition on the mutation rate:

3.17 $$\mu <\gamma_{w}\left(\frac{\kappa }{\zeta_{w}{\alpha_{w}}^{-1}+\nu_{w}}-1\right).$$

**Figure 7.**
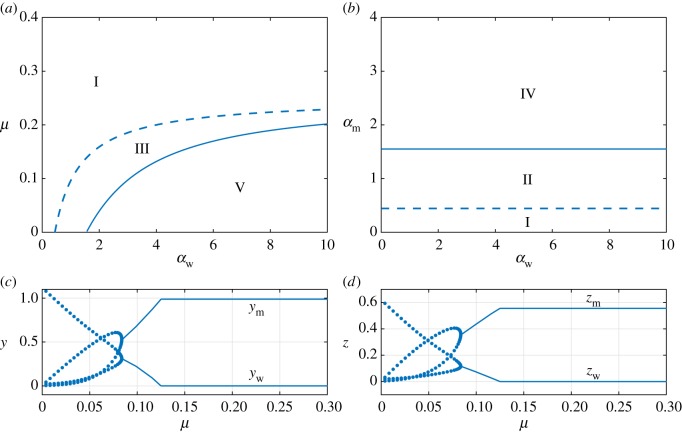
(*a*,*b*) Two-dimensional stability diagrams with a pair of bifurcation curves: transcritical bifurcations (dashed) and Hopf bifurcations (solid). Parameters, except *μ*, are given by (3.12). (*a*) We use *α*_m_ = 0.1 and consider stability diagram over parameters *α*_w_ versus *μ*. (*b*) We set *μ* = 0.5 > *μ*_*c*_ and rebuild the *α*_w_ versus *α*_m_ stability diagram. As before, Region I: stability of virus-free solution; Region II: stability of wt-free state; Region III: stability of coexistence state; Region IV: stable PO about wt-free state; Region V: stable PO about coexistence state. In the lower panels we display one-dimensional bifurcation diagrams for (*c*) infected cell and (*d*) viral strain populations versus *μ*; note, that only stable equilibria (solid curves) and stable POs (filled markers) are shown. We set *α*_w_ = 3.0 and *α*_m_ = 1.0 and track the outcompetition of the wt strains by the mutant ones at increasing *μ*, resulting in a type of error-threshold found after the oscillatory and static coexistence scenarios for *μ* satisfying (3.17).

Thus, for two strains to coexist, the mutation rate must be sufficiently small. This result supports a conjecture that excess mutation exhausts the population of the wt strain, thereby leading to a process similar to the well-known error catastrophe [70]. We would expect that a gradual increase of the mutation rate contributes to a better success of the mutant strain as the frequency of mutants generated *de novo* increases. The right-hand side of (3.17) is an increasing function of *α*_w_, reflecting the fact that a greater rate of infection by the wt virus will offset a greater mutation rate. A stability diagram in terms of the mutation rate *μ* and the wt infection rate *α*_w_ is shown in figure 7*a* for the case of *α*_m_ = 0.1 and using parameter values in (3.12). The dashed line marking the boundary between Regions I and III, and also defining the region of coexisting populations, has been obtained by replacing the inequality with equality in (3.17). As the infection rate *α*_w_ increases, the boundary between Regions I and III reaches a horizontal asymptote given by

3.18 $$\mu_{c}=\gamma_{w}\left(\frac{\kappa }{\nu_{w}}-1\right).$$

For mutation rates that satisfy *μ* < *μ*_*c*_, Regions III and V can be entered from Region I by increases in the infection rate, promoting coexistence. However, for *μ* > *μ*_*c*_, only the wt-free state can occur. Hence, equation (3.18) defines a critical, finite mutation rate at which coexistence no longer becomes possible due to extinction of the wt virus due to the outcompetition by the mutant strains. To explore this in more detail, we have repeated the stability diagram shown in figure 4*b* using a value of *μ* = 0.5 > *μ*_*c*_. The stability diagram changes drastically, becoming that shown in figure 7*b*, and contains only three regions: I, II and IV. The stability diagrams in figure 4*b* and 7*b* are linked through the fact that as the mutation rate increases, the DBT bifurcation shifts to the right, eventually tending to infinity as the critical value is approached.

Finally, figure 7*c*,*d* illustrates how the equilibrium populations of infected cells and viral strains change with increasing mutation rate *μ*. Specifically, we have used *α*_w_ = 3.0 and *α*_m_ = 1.0, along with the parameters in (3.12), in both panels. As *μ* increases beyond the point where (3.17) is satisfied, the population of mutants (virions and infected cells) outcompetes the wt populations (figure 7*c*,*d*). This phenomenon is similar to the error threshold defined in quasi-species theory. Here we show that mutation is not only involved in this shift, but also depends (at the infection-cell level) on virulence, burst size and multiplicity of infection.

Throughout the previous analyses, we have assumed that the wt strain of the virus mutates and the new strain has no chance of mutating exactly into the original strain. Although it is highly unlikely, the full picture of the proposed model would require a consideration of a possibility for backwards mutation, especially when modelling phenotypic traits. We therefore replace (2.4b,c) with

3.19*a* $${\dot{y}}_{m}=\alpha_{m}z_{m}x+\mu_{w}y_{w}-\mu_{m}y_{m}-\gamma_{m}y_{m}$$

and

3.19*b* $${\dot{y}}_{w}=\alpha_{w}z_{w}x-\mu_{w}y_{w}+\mu_{m}y_{m}-\gamma_{w}y_{w}.$$

We begin our investigation of the role of backwards mutation by re-building the two-dimensional bifurcation diagram and stability diagrams shown in figure 4 using the parameters in (3.12). The mutation rate of the wt virus is set to *μ*_w_ = 0.1 and we consider two values of the mutation rate of the mutant virus. First, we take *μ*_m_ = 10^−3^ ≪ *μ*_w_, so that the new form of mutation can be considered as a small perturbation to the original system of equations (2.1). Then, we equate the mutation rates of both strains and set *μ*_m_ = 0.1.

**Figure 8.**
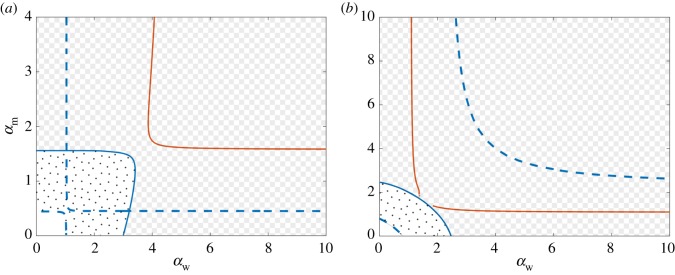
Co-dimension two bifurcation diagrams in terms of the wt and mutant virus infection rates *α*_w_ and *α*_m_ when backwards mutation is possible. The mutation rates are (*a*) *μ*_m_ = 10^−3^ and *μ*_w_ = 10^−1^ and (*b*) *μ*_m_ = 10^−1^ and *μ*_w_ = 10^−1^. All other parameters are given by (3.12). Dashed and solid lines correspond to transcritical and Hopf bifurcations, respectively. Blue denotes biologically feasible equilibria undergoing the bifurcations (all components non-negative), while colour orange denotes bifurcations of equilibria with negative components. The non-patterned, polka-dot and checkerboard regions correspond to virus-free, stationary coexisting, and oscillatory coexisting states, respectively.

The two-dimensional bifurcation diagrams in figure 8*a*,*b* reveal that backwards mutation results in several important changes to the dynamics. Importantly, the behaviour of the system can be described using three main states: the virus-free state (analogous to Region I and displayed in white in figure 8), stationary coexistence all populations (polka dot pattern in figure 8, analogous to Region III), and oscillatory coexistence of all the populations (checkerboard pattern in figure 8, analogous to Region V). Furthermore, the DBT and the DZH bifurcations have vanished, the latter of which implies that the curve of TPO bifurcations no longer exists either. The vertical and horizontal lines of transcritical bifurcations defined by $R_{0}^{w}=1\;\mathrm{and}\;R_{0}^{m}=1$, which intersected at right angles in figure 4*a*, have now merged into two separate branches that do not intersect. The curves of Hopf bifurcations, which also intersected in the case of uni-directional mutation, have also merged into two distinct non-intersecting branches. Finally, the transcritical bifurcation between the wt-free and coexistent states ***v***_2_ and ***v***_3_ has vanished as well. The resulting stability diagram, shown in figure 8 by patterns, is now considerably simpler and involves only analogues of Regions I, III and V. Thus, the only virus-persistent states are those in which both virus strains coexist. The wt-free state can no longer occur due to the creation of the wt virus through backwards mutation.

## 4. Conclusion

In this paper, we studied a mathematical model of population dynamics of two viral strains infecting a population of the same cell type. Unlike previous models of parasite–host-like interaction, we consider the emergence of a second strain by mutation from a single strain introduced into the environment. The mechanism of virus replication forces the consideration of two types of infected cells, one for each viral strain, in the model. We analysed the system of differential equations and its solutions. By studying parameters space, we identified five different regions, each characterized by distinct dynamics: (Region I) the virus-free state which maximizes the population of host cells, (Region II) the stationary existence of mutant virus with nonzero cell populations supporting it, (Region III) the stationary persistence of both viral strains with nonzero cell populations, (Region IV) the oscillating existence of the mutant virus and corresponding cell populations but extinct wt virus population, and finally, (Region V) the oscillating coexistence of all populations.

The population of the mutant-type virus exists as long as the mutation rate of the wt strain is positive. The broken symmetry between the wt and mutant strains is clear from the stability diagrams based on infection rates. From our results, we observe that survival of the wt virus is essential for coexistence. However, the growth of the wt virus population jeopardizes its persistence in the system by creating its own competitor: the mutant-type virus. Moreover, the larger the mutation rate, the greater the infection rate of the original wt strain should be in order to remain in the system. Interestingly, we have found a maximum—critical value—of the mutation rate in the context of the model which allows for the persistence of the wt strain and hence coexistence. Values of the mutation rate that are higher than the critical value make coexistence impossible even for the most infectious wt strain. Furthermore, our model shows that the concept of the error threshold may not be considered as a one-parameter-driven effect, i.e. based solely on the mutation rate [69,70]. The critical value of mutation is shown here to be proportional to the virulence and burst size of the wt virus and proportionally inverse to its multiplicity of infection. Hence, our results extend the phenomenon of the error threshold at the infected cell population level.

## Data accessibility

MATCONT source files, output data and MATLAB files used to create the figures in the manuscript are available from the Dryad Digital Repository: https://doi.org/10.5061/dryad.56g7v08 [65].

## Appendix A. Further study of degenerate Bogdanov–Takens and zero-Hopf bifurcations

Normal form theory provides a means of predicting the local dynamics that occur near a given bifurcation. This is possible because the local structure of a bifurcation is model independent and thus can be immediately determined from pre-existing analyses of simpler systems of equations. The simplest system of equations that completely captures the dynamics of a particular bifurcation is called the normal form. The conducted numerical studies on the system (2.4) revealed that the Bogdanov–Takens and zero-Hopf bifurcations are non-standard in the sense that the local dynamics do not agree with those predicted by their normal forms. Such a mismatch can occur when one of the terms in the normal form equations vanishes due to its coefficient being equal to zero, implying that an extended system of equations needs to be considered. We now examine the normal forms of the Bogdanov–Takens (BT) and zero-Hopf (ZH) bifurcations in order to rationalize the discrepancies between the two-dimensional bifurcation diagram shown in figure 4 and those predicted from normal form theory.

The local bifurcation diagram near a BT point can be determined from the normal form equations given by (e.g. Kuznetsov [66, §8.4])

A 1*a* $${\dot{\xi }}_{0}=\xi_{1}$$

and

A 1*b* $${\dot{\xi }}_{1}=\beta_{1}+\beta_{2}\xi_{0}+a_{2}\xi_{0}^{2}+b_{2}\xi_{0}\xi_{1},$$

provided that the coefficients *a*_2_ and *b*_2_ are not equal to zero, *a*_2_*b*_2_ ≠ 0. The quantities *β*_0_ and *β*_1_ in equations (A 1) play the role of bifurcation parameters, with *β*_0_ = *β*_1_ = 0 corresponding to the BT point. The bifurcation diagram of system (A 1) consists of curves of saddle-node and Hopf bifurcations, neither of which were detected in the local analysis of the model analysed in this article (2.4) near the BT point, as well as curves of global bifurcations involving homoclinic orbits. The procedure outlined by Kuzetnsov [72] enables the normal form coefficients to be related to the model studied here. Remarkably, the coefficient *a*_2_ can be calculated analytically and is found to be equal to zero for all parameter combinations. Thus, the BT bifurcation occurring in our model is always degenerate and the local dynamics will be different from those of equation (A 1).

The vanishing of *a*_2_ can be rationalized in terms of the number of equilibria at the degenerate Bogdanov-Takens (DBT) point. An equilibrium analysis shows that (A 1) will only have two equilibria at the DBT point *β*_0_ = *β*_1_ = 0 if *a*_2_ ≠ 0; however, the model under study has three. Thus, *a*_2_ = 0 is needed to capture the triple equilibrium at the DBT point in our model. This also suggests that higher-order terms must be included in (A 1) in order for it to capture the DBT bifurcation in the model investigated here. While an extended system of normal form equations for degenerate BT bifurcations is proposed in Kuznetsov [72], it cannot capture the transcritical bifurcations found in the original model, suggesting that an alternative form is required. However, due to the simple nature of the local dynamics near the degenerate BT point shown in figure 2, which can be obtained analytically from the full model (2.4), we do not pursue this point further.

The Poincaré normal form of a ZH bifurcation can be written as [66, §8.5]

A 2*a* $$\dot{v}=\gamma (\beta )+\frac{1}{2}G_{200}(\beta )v^{2}+G_{011}(\beta )|w{|}^{2}+\frac{1}{6}G_{300}(\beta )v^{3}+G_{111}(\beta )v|w{|}^{2}$$

and

A 2*b* $$\dot{w}=\Lambda (\beta )w+H_{110}(\beta )vw+\frac{1}{2}H_{210}(\beta )v^{2}w\frac{1}{2}H_{021}(\beta )w|w{|}^{2},$$

where *β* = (*β*_1_, *β*_2_) is a vector of bifurcation parameters. Analyses of the normal form equations for the ZH bifurcation show that local dynamics depend on the values of the normal form coefficients *H*_*ijk*_ and *G*_*ijk*_. A common feature between the various cases is that the ZH bifurcation occurs at the tangential intersection of curves of saddle-node and Hopf bifurcations. However, figure 4*a* shows that the zero-Hopf bifurcation in our model lies at a transversal intersection of curves of transcritical and Hopf bifurcations. Furthermore, none of the normal forms predict that a curve of TPO bifurcations should emanate from a ZH point. The calculation of the three normal form coefficients for the DZH bifurcation is rather involved and must be performed numerically. We find that the normal form coefficient *G*_011_(0) = 0 across a range of parameter values, indicating the ZH bifurcation is degenerate. The normal form equations for degenerate ZH bifurcations are not well known and previous studies have focused on different cases [73,74]. Tigan *et al.* [75] showed that a degenerate ZH bifurcation with *G*_011_(0) = 0 occurs in a Rössler-type system and used averaging theory to detect periodic orbits, leaving the structure of global bifurcations unresolved. We believe, as far as we know, that this is the first time a curve of TPO bifurcations has been observed to emanate from a degenerate ZH bifurcation of this type. We leave determining the corresponding normal form as an interesting area of future work.
